# Cochlear duct length in Pakistani cochlear implant recipients gender, age and side association: A Radiological Measure

**DOI:** 10.12669/pjms.40.1.7426

**Published:** 2024

**Authors:** Zahra Sarwar, Jawwad Ahmed, Ghulam Saqulain, Muhammad Iqbal Javed Khan

**Affiliations:** 1 Dr. Zahra Sarwar, MBBS. Post-Graduate FCPS Trainee ENT Department, KRL Hospital, Islamabad, Pakistan; 2 Dr. Jawwad Ahmed, FCPS. Implant Surgeon, Department of Otorhinolaryngology Capital Hospital PGMI, Islamabad, Pakistan; 3 Dr. Ghulam Saqulain, FCPS. Head of Department/ Professor of Otorhinolaryngology Capital Hospital PGMI, Islamabad, Pakistan; 4 Dr. Muhammad Iqbal Javed Khan, FRCS. Consultant Otologist and Skull Base Surgeon, Bradford Teaching Hospitals NHS Foundation Trust, United Kingdom

**Keywords:** Computed tomography scan, Cochlear duct length, Cochlear Implant, Gender

## Abstract

**Objectives:**

To analyze the gender, age and side association of cochlear duct length in Pakistani-Asian cochlear implant recipient population based on computed tomography imaging study.

**Methods:**

Current study retrospectively studied charts of cases who underwent cochlear implantation at the Department of Otolaryngology & Auditory Implant Centre, Capital Hospital Islamabad, over a period of two years from 1st May 2017 to 30th April 2019. These included 200 cases of both genders and of any age. In addition to basic demographic data, computed tomography findings of the temporal bone were utilized to measure the cochlear duct length. Data was analyzed using SPSS Version 23.

**Results:**

Study revealed a mean Cochlear duct length of 29.935±2.173mm (range: 25.12 to 37.60) with significant (p<0.001) association with gender with longer cochlear duct in males compared to females on right (30.50±2.384 vs. 29.36±1.887) and on left side (30.50±2.236 vs.29.32±1.935). However, no significant difference was noted for side with slightly longer cochlear duct on the right side compared to left (29.95±2.224 vs.29.92±2.171). Also, no significant association with age was noted with p=0.578 & p=0.824 for right and left side respectively.

**Conclusion:**

Pakistani population is characterized by a short mean CDL of 29.935±2.173 mm with significant association (p<0.001) with gender with longer cochlear duct length in males; and side with larger CDL on right side. However, no significant association with age was noted.

## INTRODUCTION

Congenital Hearing Loss (HL) is quite prevalent in developing countries with a self-reported HL of 13/1000 in Pakistan with 15% suffering from profound HL.^1^ Cochlear implantation being the state of the art treatment for profound HL, has become established in Pakistan over the last few decades and following the establishment of first public cochlear implant center of the country in Islamabad attention has been drawn to the anatomical variations of the inner ear,^2^ being of significant importance in surgical planning and predicting prognosis. With advances in imaging technology, detailed morphologic description of cochlear structures is now possible allowing large studies while evaluating cases for Cochlear Implantation using automatic tracing allowing vertical as well as cochlear duct size measurements.^3^ Cochlear duct length measurement is necessitated for selection of right sized implant electrode array and frequency map customization and has been facilitated with the advancement in Computed Tomography (CT) imaging technology with measurement focus on lateral wall, reconstruction of cochlear shape based on spiral coefficients using 3D reconstruction being highly reliable method.^4^ With same density of outer and inner hair cells, cochlear duct length (CDL) is considered to have influence on frequency resolving status of the ear.^5^ Differences in CDL have been reported in different populations.^6^ As noted in a review by Zanon & Martini, in otolaryngology, sex difference and its impact is a uncharted area with studies in some specific areas nearly nonexistent, while bias exists in others. Dissimilarities exist between different genders in terms of epidemiology, pathophysiology, clinical features, treatment and response, hence requiring research since this gender issue has to act as a filter through which evidence based practice should pass.^7^ A study by Miller JD involving 148 skulls from different sources revealed that on an average cochlear duct length was 3.36% longer in males compared to females.^5^ Anatomic variations of the audio-vestibular system with gender may be influenced by hormonal or physiological influences and can result in variations in clinical outcomes, hence this is a topic required to be investigated.^8^ Measuring of CDL is of importance for further development in the field of cochlear implantation and hence research is emphasized.^9^ According to Alanazi & Alzhrani is essential to measure the CDL in different population.^6^

Rich genetic heterogeneity as regards HL, makes Pakistani population more suitable for research.^10^ This along with a high prevalence of congenital HL and need of research in the field regarding cochlear duct length in different strata of population and establishment of public sector cochlear implant center having necessary infrastructure facilitated research & compelled the authors to conduct this study to analyze the gender, age and side association of cochlear duct length in Pakistani-Asian Population based on computed tomography imaging study. This study is of significant importance as it might be of clinical implications on electrode array insertion and design, to avoid frequency-to-place mismatch, hence important for implant programs and for recommendations of any alterations if required.

## METHODS

This study retrospectively reviewed charts of cases who underwent cochlear implantation for SNHL over a period of two years 1st May 2017 to 30th April 2019. Study was conducted at Capital Hospital PGMI in the department of Otolaryngology & Cochlear Implant Centre. Sample included 200 cases of both genders with no age limit. Cases with ear anomalies were excluded from the study. After collection of demographic data, high resolution computed tomography (HRCT) scans of temporal bone of the operated cases were reviewed to measure the cochlear duct length.

### Ethical Approval

Study was conducted after obtaining ethical approval of ethical research committee of Capital Hospital PGMI, Islamabad vide Reg No. 2021-02-004.

HRCT was utilized to measure the CDL utilizing the formula proposed by Alexiades et al.^11^ CDL=(4.16A)-4. Cochlear Length (A) (CLA), was measured from the center of round window to the most distant point on the wall of cochlea on the opposite site i.e., helicotrema, which passed through modiolus. This was carried out in minimum intensity projection mode of reformatted image 12.

### Statistical Analysis

Data was entered and tabulated in Microsoft Excel Worksheet and later analyzed statistically using Statistical Package for the Social Sciences (SPSS) Version-23. Results were presented utilizing frequencies, percentages, mean, and standard deviation. T-Test & ANOVA statistics were utilized to determine any significant difference between groups. P<0.05 was considered significant.

## RESULTS

**Fig.1:**
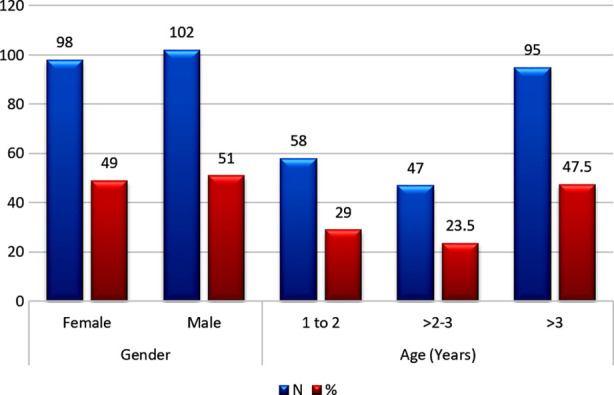
Demographic characteristics of Sample population (N=200).

**Fig.2:**
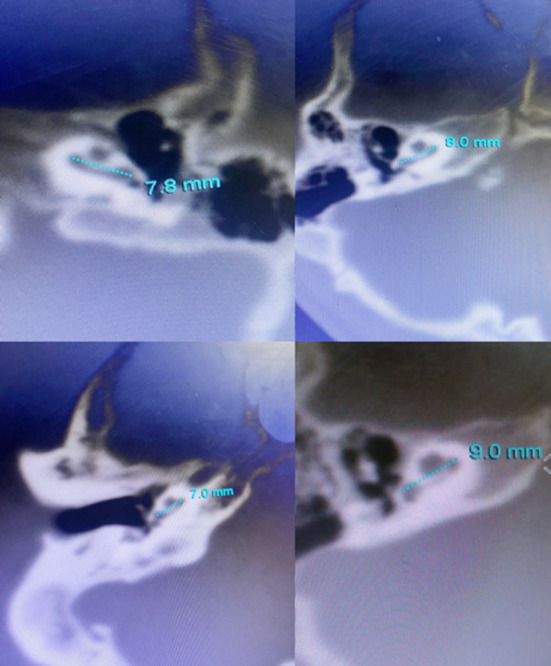
Computed tomography images of temporal bone showing with Cochlear Length ‘A’ (CLA) measurement of few cases.

Current study with sample of N=200 cochlear implant candidates revealed 102(51%) males and 98(49%) females with majority 95(47.5%) being above three years of age (Fig.1). In current population for the right ear the mean CLA was 8.16±0.54 mm (7-10) with few CT images showing CLA measurement in Fig.2, and CDL 29.95±2.22 mm (25.12 to 37.60) and for the left ear CLA was 8.16±0.52 (7-10) and CDL 29.92±2.17mm (25.12- 37.60) (Table-I).

**Table-I:** Descriptive statistics for Cochlear Length ‘A’ (CLA) and Cochlear Duct Length (CDL) (n=200).

Type of Length	Length	Mean± SD (mm)	Minimum (mm)	Maximum (mm)
Cochlear Length ‘A’	Right Ear	8.16±0.54	7.00	10.00
	Left Ear	8.16±0.52	7.00	10.00
	Total	8.157±0.53	7.00	10.00
Cochlear Duct Length	Right Ear	29.95±2.22	25.12	37.60
	Left Ear	29.92±2.17	25.12	37.60
	Total	29.935±2.173	25.12	37.60

To see the between group difference in Cochlear Duct Length in Pakistani population (Table-II), t-test and ANOVA statistics were utilized, which revealed significant (p<0.001) gender association of cochlear duct length with longer cochlear duct in males compared to females on right (30.50±2.384 vs. 29.36±1.887) and on left side (30.50±2.236 vs. 29.32±1.935). However, no significant difference was noted for different age groups with p=0.578 & p=0.824 for right and left side respectively. Also, there was no significant association of CDL (p=0.656) with slightly longer cochlear duct on the right side compared to left (29.95±2.224 vs.29.92±2.171).

**Table 2:** Demographic Variable Versus Cochlear Duct Length cross tabulation. T-Test/ Anova Statistics (N=200).

Variable Detail			Cochlear Duct Length			T-Test/ ANOVA Statistics

Variable	Cochlear Duct	Group (n)	Mean± SD (mm)	Std. Error Mean (mm)	t/f	P-value
Gender	Right side	Male (102)	30.50±2.384	0.236	3.735	0.000
		Female (98)	29.36±1.887	0.191		
	Left side	Male (102)	30.50±2.236	0.221	3.988	0.000
		Female (98)	29.32±1.935	0.195		
Age Group (Years)	Right side	01-02 (58)	29.85±2.127	0.279	0.549	0.578
		>2-3 (47)	29.72±1.785	0.260		
		>3 (95)	30.11±2.472	0.254		
	Left side	01-02 (58)	29.85±2.127	0.279	0.194	0.824
		>2-3 (47)	29.81±1.864	0.272		
		>3 (95)	30.02±2.348	0.241		
Side	Right side	200	29.95±2.224	0.157	0.446	0.656
	Left side	200	29.92±2.171	0.154		

## DISCUSSION

Current study with 102(51%) males and 98(49%) females with majority 95(47.5%) of population being above three years of age revealed a mean CLA of 8.16±0.52mm (7.00-10.00) and mean CDL of 29.935±2.173mm (25.12-37.60). Similarly, Grover et al. in an Indian study reported mean CLA as 8.12mm.^13^ Similarly two other Indian studies revealed a mean CDL of 30.7mm (27.6-33.4) by Singh A et al.^12^ and 29.8 mm (28-34.3) by Grover M et al.,^14^ indicating a small CDL in Indo-Pak subcontinent while a slightly longer CLA of 8.75±0.31mm and CDL of 32.45±1.31 (3.01-34.83) has been reported by Zahara D et al. for Indonesian population.^15^ Even larger cochlea have been reported from Western countries with Spiegel JL et al. in a German study reported a broad range of CDL with every cochlea measuring more than 31 mm with a mean CDL of 36.2±1.8 mm.^16^ Erixon E et al. in a European study reported mean CDL of 42.2±1.86 (37.6 - 44.9mm)^17^ and Ketten DR et al. revealed CLA of 33.01±2.31,^18^ indicating a small CDL in Indo-Pak subcontinent compared to others especially western countries.

Current study population revealed no significant association (p=0.656) of CLA and CDA between side of ear with mean CLA of 8.16±0.54mm (7-10) and CDL 29.95±2.22 (25.12-37.60) for the right side and for the left ear CLA was 8.16±0.52mm (7-10) and CDL 29.92±2.17mm (25.12- 37.60). Similarly an Indian study with large sample size of 129, by Grover M et al. revealed a slight difference with mean cochlear CLA for right ear 8.10 mm (7.7- 9.2) and left ear 8.14 mm (77-9.0).^14^ In contrast in an Indian study by Singh A et al. no significant (p=0.52) association between two sides was noted with CDL being 30.5±1.59mm on the right and 30.8±1.74mm on the left side.^12^ Similarly no significant association with side was noted in German studies,^16^ and in a Saudi study by Khurayzi T et al. reported revealed no significant difference (p=0.704) with CDL for right 8.45 and left 8.42 mm side.^19^

Current study revealed significant (p<0.001) gender association of cochlear duct length with longer cochlear duct in males compared to females on right (30.50±2.384 vs 29.36±1.887) and on left side (30.50±2.236 vs 29.32±1.935). Similarly difference was reported in a Chinese study with CLA of 9.04+0.3 mm in males and 8.80+0.4 mm in females.^20^ Also Alanazi et al. & Alzhrani et al. in a Saudi study reported a significant (p=0.003) gender difference in the CDL with overall mean length of 32.27±2.48 mm for males and 31.51±2.75 mm for females. They also reported significantly (p<0.001) longer CDL of 32.199±2.869 mm on the left side and shorter 31.565±2.785 mm on the right side.^6^

Similarly, significant (p=0.037) gender difference was noted by Spiegel JL et al in a German study with a longer CDL in males (36.5 ±0.2mm) compared to females (35.8±0.3mm).^16^ Wurfel W et al. in a European study also reported significant difference (p<0.001) gender^21^ & a Saudi study also reported significant gender difference with of CDL 8.54 in males and 8.34 in females (P=0.016).^19^ Similarly another study reported significantly longer CDL in males (34.5mm) compared to females (33.3mm). 22

Though age is reported to have significant association with inner ear volume,^23^ however current investigation did not reveal any significant difference of CDL for different age groups with p=0.578 & p=0.824 for right and left side respectively. In conformity to our study in a European study by Wurfel W et al. also noted no significant difference with p=0.301.^21^

Current study has filled the gap in literature as regards CDL length in local population which is significantly shorter than Western population and has gender and side association being longer in males and on right side. This has clinical implications on electrode array insertion and design, hence important for implant programs and for recommendations for alterations.

### Recommendation

Research to evaluate whether CDL can predict speech outcome and relation to congenital Sensorineural hearing loss are required.^24^

### Limitations

Study did not take into account the head diameter and height of the patient, which could also impact the CDL.

## CONCLUSIONS

Pakistani population is characterized by a short mean CDL of 29.935±2.173 mm with significant association (p<0.001) with gender with longer cochlear duct length in males; and side with larger CDL on right side. However, no significant association with age was noted.

### Authors` Contribution:

**ZS** was responsible for data collection.

**JA** did the design of research and interpretation and analysis of results.

**GS** did the literature review, writing of manuscript and was responsible for integrity of research.

**MIJK** did the methodology & critical revision of the article.
